# Smoking Behavior and Related Lifestyle Variables among Physicians in Fukuoka, Japan: A Cross Sectional Study

**DOI:** 10.2188/jea.12.199

**Published:** 2007-11-30

**Authors:** Akihiko Kaetsu, Tetsuhito Fukushima, Masaki Moriyama, Takao Shigematsu

**Affiliations:** 1 Department of Hygiene, Faculty of Medicine, Tottori University.; 2 Department of Public Health, School of Medicine, Fukuoka University.; 3 Professor Emeritus, Fukuoka University.

**Keywords:** physician, prevalence of smoking, lifestyle, cross-sectional study

## Abstract

A cross-sectional survey of the entire membership of the Fukuoka Prefecture Medical Association was conducted in 1983 using a self-administered questionnaire. In this investigation the actual prevalence of smoking among physicians and the relationship between their smoking habits and living habits were studied. The study subjects were divided into two groups: those who smoked (1,737 men and 17 women), and those who did not currently smoke (2,267 men and 169 women). It was realized that there were many who were currently non-smokers among women, subjects with a high body mass index, those with heart disease, those without peptic ulcers, those who underwent health check-ups regularly, those accustomed to an early bedtime, those who were not aware of mental stress, those who took regular exercise, those who consumed plenty of fresh vegetables, yellow and green vegetables and fruit, those who did not consume Japanese pickles, coffee or green tea, and those who drank alcohol only occasionally and only in small amounts. The results of this study suggested the possibility that physicians who were smokers were a group who smoked little and could easily stop smoking. Moreover, non-smoking physicians were found to have a healthier lifestyle than those who smoked. It was considered that, in developing a smoking cessation program for physicians, it is important for them to establish more health-conscious lifestyles.

## INTRODUCTION

In Japan, mortality due to lung cancer is increasing, and since 1998, it has ranked as the top form of cancer in both sex (males and females combined). Moreover, these increases are expected to continue in the future^1^^)^.

In the United States of America, however, decreases in both the incidence and mortality due to lung cancer have been seen since the early 1990s^2^^)^. The proportion of smokers among men in Japan has shown a tendency to fall since the 1970s, whereas, among women, no great change has been noted. The overall total for both sexes in Japan has tended to decline, but is still the highest of any advanced country^3^^,^^4^^)^.

It has been reported that, when a physician, in his/her daily medical practice, gives the advice to quit smoking, the proportion of smokers receiving such advice who manage successfully to give up smoking is increased^5^^)^. However, it is also reported that whether or not a physician will advise a patient to stop smoking depends very much on the personal smoking status —smoker or non-smoker — of that particular physician^6^^)^. Lowering the prevalence of smoking among physicians is likely to be an important measure in lowering the prevalence of smoking among the Japanese as a whole.

In the past, various cross-sectional studies have been carried out on smoking among physicians in Japan. The results of these studies seem to indicate that the prevalence of smoking among Japanese physicians is on the decline^7^^-^^12^^)^. But the prevalence of smoking among Japanese physicians is still extremely high in comparison with the corresponding rates in the U.S.A. and the United Kingdom^13^^-^^15^^)^.

Although reports have appeared on the relationship between the smoking habits of Japanese physicians and their medical knowledge and beliefs^6^^-^^12^^)^, we have been able to find no report on how lifestyle and smoking are related.

If the characteristics of physicians’ lifestyles that affect their smoking status can be discovered, it should be possible to devise a smoking cessation program for physicians.

The present study, by clarify some characteristics of smokers and non-smokers, aimed to reveal a relationship between the smoking status of physicians and their lifestyles.

## MATERIALS AND METHODS

In June 1983, a lifestyle survey was conducted on the entire complement of physicians registered as members of the Fukuoka Prefecture Medical Association as of April 1, 1983 — a total of 4,980 physicians (4,755 men and 225 women). The lifestyle survey employed a self-administered questionnaire in which the subjects were requested to answer in their own names. The distribution and collection of the questionnaires were carried out with the aid of the various local branches of the Medical Association, and 4,232 responses were obtained: 4,042 from male, and 190 from female, physicians.

For the present study, total counts were made, classified by sex, age, and smoking habits, for 4,190 physicians (4,004 men and 186 women). Valid responses were provided by 84.2% of the men and by 82.7% of the women (84.1% overall).

The questionnaire consisted of the following categories of questions: individual characteristics, physical condition, conditions of work, recreation, exercise habits, and food and beverage intake. The factors considered in the study were selected from those in the questionnaire, with reference to earlier research works^16^^-^^28^^)^.

As individual characteristics, sex, age, and body mass index (BMI) were selected. Regarding physical condition, five items were chosen: subjective assessment of condition of health, current heart disease, current peptic ulcer disease, frequency of health check-ups, and customary medication. In relation to conditions of work also, five items were used: owner-practitioner or employee, specialty of medical practice, number of working days, number of patient consultations, and consulting hours. For recreation, there were four items: early or late bedtime, duration of sleep, and awareness of physical fatigue and mental stress, and for exercise, current habits of physical exercise. As for food intake, seven items were observed: the daily consumption levels of miso soup, Japanese pickles, bread, milk, fresh vegetables, yellow and green vegetables, and fruit. Finally, regarding beverages, four items were selected: the daily consumption of coffee and green tea, the frequency of alcohol intake, and the amount of alcohol intake.

In relation to smoking status, in the questionnaire, the subjects were requested to choose one of the following three categories: never smoked (non-smoker), used to smoke but no longer do (past smoker), and currently smoke (current smoker). Since the study focused on understanding the characteristics of the lifestyles of current smokers and current non-smokers, two categories were used in the analysis: current smokers, and non-smokers and past smokers combined. The relationships between the smoking status and the 29 questionnaire variables of characteristics and lifestyle items were then studied.

First, the relationship between each item and smoking status was examined using the chi square test. Each lifestyle factor for which a significant difference was found was employed as an independent variable in a univariate unconditional logistic regression analysis where the dependent variable was smoking status, and the odds ratio (OR) and the 95% confidence intervals (95%CI) were calculated. In the step-wise multivariate logistic regression analysis, the factors that showed a significant correlation with smoking status in the univariate unconditional logistic regression analysis were used as the independent variables, after being adjusted for age and sex, and variables with a significant relation to smoking habits were selected. The odds ratios were sat greater than 1.0, when it contributed to non-smoking. All statistical calculations were carried out by the SAS statistical software package, version 6.12 (SAS Institute, Cary, NC).

## RESULTS

Age and sex distribution of respondents by conditions of work, owner-practitioner and employed physician was given in Table 1. Among them, 96% were male physicians. The most common age group was in 50 years of age in males and 60s in females, respectively. The proportion of owner-practitioners were 76% in males and 59% in females.

**Table 1.** Distribution of conditions of work by sex and age.

Sex Age	Owner	Employed	Total ( % )	
Male				
-40 years	168	154	322	( 8.0 )
40-49 years	579	213	792	( 19.8 )
50-59 years	1,216	256	1,472	( 36.8 )
60-69 years	736	180	916	( 22.9 )
70+ years	345	157	502	( 12.5 )
Total	3,044	960	4,004	(100.0 )

Female				
-40 years	13	23	36	( 19.4 )
40-49 years	17	14	31	( 16.7 )
50-59 years	24	19	43	( 23.1 )
60-69 years	40	11	51	( 27.4 )
70+ years	16	9	25	( 13.4 )
Total	110	76	186	(100.0 )

Owner: Owner of their own hospital or clinic

Employed: Employed physician

Smokers accounted for 43% of male physicians, a far higher proportion than the 9% of female physicians. As shown in Table 2, the proportions by age group were 48% for those under 40 years of age, 40% for those in their 40s, 46% for those in their 50s, 42% for those in their 60s, and 31% for those in their 70s and above. When the figures for owner-practitioners and employed physicians were examined separately, it was seen that the proportions of smokers among the former were greater than those among the latter in both men and women (not significant). The age-adjusted smoking proportion of physicians using National survey of circulatory disorders 1980^29^^)^ as standard were 45% in males and 9% in females.

**Table 2.** Smoking prevalence by sex and age in conditions of work.

Sex Age	Owner			Employed			Total		
		
	N	Smoker	( % )	N	Smoker	( % )	N	Smoker	( % )
Male									
-40 years	168	83	( 49.4 )	154	83	( 53.9 )	322	166	( 51.6 )
40-49 years	579	249	( 43.0 )	213	78	( 36.6 )	792	327	( 41.3 )
50-59 years	1,216	569	( 46.8 )	256	117	( 45.7 )	1,472	686	( 46.6 )
60-69 years	736	325	( 44.2 )	180	71	( 39.4 )	916	396	( 43.2 )
70+ years	345	110	( 31.9 )	157	52	( 33.1 )	502	162	( 32.3 )
Total	3,044	1,336	( 43.9 )	960	401	( 41.8 )	4,004	1,737	( 43.4 )
Age-Adjusted			( 44.6 )			( 43.3 )			( 44.6 )

Female									
-40 years	13	2	( 15.4 )	23	3	( 13.0 )	36	5	( 13.9 )
40-49 years	17	1	( 5.9 )	14	0	( - )	31	1	( 3.2 )
50-59 years	24	3	( 12.5 )	19	2	( 10.5 )	43	5	( 11.6 )
60-69 years	40	5	( 12.5 )	11	0	( - )	51	5	( 9.8 )
70+ years	16	1	( 6.3 )	9	0	( - )	25	1	( 4.0 )
Total	110	12	( 10.9 )	76	5	( 6.6 )	186	17	( 9.1 )
Age-Adjusted			( 11.0 )			( 5.9 )			( 9.1 )

Both combined									
-40 years	181	85	( 47.0 )	177	86	( 48.6 )	358	171	( 47.8 )
40-49 years	596	250	( 41.9 )	227	78	( 34.4 )	823	328	( 39.9 )
50-59 years	1,240	572	( 46.1 )	275	119	( 43.3 )	1,515	691	( 45.6 )
60-69 years	776	330	( 42.5 )	191	71	( 37.2 )	967	401	( 41.5 )
70+ years	361	111	( 30.7 )	166	52	( 31.3 )	527	163	( 30.9 )
Total	3,154	1,348	( 42.7 )	1,036	406	( 39.2 )	4,190	1,754	( 41.9 )
Age-adjusted			( 43.2 )			( 40.3 )			( 42.6 )

Owner: Owner of their own hospital or clinic, Employed: Employed physician, N: No. of subjects

Age-adjustment was calculated by direct method using National survey on circulatory disorder 1980 as standard.

The relationships between the 29 variables and smoking status (non-smoking) were shown in Table 3. In the chi square test (p < 0.05), significant relationships were observed for 22 factors. In the univariate unconditional logistic regression analysis, the following 15 of those 22 factors showed significantly higher proportions of non-smokers than in each basic category: (1) women, (2) age 60 years and over, (3) BMI 22 and over, (4) the presence of peptic ulcer disease, (5) having medical check-ups, (6) a work week of less than 5 days, (7) bedtime before midnight, (8) no awareness of physical fatigue, (9) no awareness of mental stress, (10) taking physical exercise, (11) consumption of bread on a daily basis, (12) daily consumption of milk, (13) daily consumption of fresh vegetables, (14) daily consumption of yellow and green vegetables, and (15) daily consumption of fruit. By contrast, in the following 7 of those 22 factors, there were significantly fewer non-smokers compared to each basic category: (1) the presence of heart disease, (2) owner-practitioner, (3) consumption of Japanese pickles on a daily basis, (4) daily consumption of one cup of coffee or (5) of green tea, (6) daily or almost daily alcohol consumption, and (7) consumption of one alcoholic (180 ml) drink or more a day.

**Table 3.** The relationship between lifestyle variables and smoking status (non-smoking).

Variables	N	Non-smoker	(%)	p for chi-square	Odds Ratio	95%CI
1. Personal characters						
Sex						
Male	4,004	2,267	56.6		1.00	
Female	186	169	90.9	p= 0.001	7.62	4.61 - 12.59
Age						
-50 years	1,181	682	57.7		1.00	
50-59 years	1,515	824	54.4		0.87	0.75 - 1.02
60+ years	1,494	930	62.2	p= 0.009	1.21	1.03 - 1.41
Body Mass Index						
Under 22	1,480	820	55.4		1.00	
22 and over	2,710	1,616	59.6	p= 0.008	1.19	1.05 - 1.35
2. Physical conditions						
Subjective condition of health						
Not good	1,037	626	60.4		1.00	
Good	3,153	1,810	57.4	p= 0.094	0.89	0.77 - 1.02
Heart disease						
No	3,997	2,300	57.5		0.57	0.41 - 0.78
Yes	193	136	70.5	p= 0.001	1.00	
Peptic ulcer						
No	4,114	2,408	58.5		2.42	1.51 - 3.87
Yes	76	28	36.8	p= 0.001	1.00	
Health check-ups						
No	1,727	947	54.8		1.00	
Yes	2,463	1,489	60.5	p= 0.001	1.26	1.11 - 1.43
Customary medication						
No	2,110	1,202	57.0		1.10	0.98 - 1.25
Yes	2,080	1,234	59.3	p= 0.122	1.00	
3. Conditions of work						
Owner or employed						
Employed	1,036	630	60.8		1.00	
Owner	3,154	1,806	57.3	p= 0.045	0.86	0.75 - 1.00
Specialty of medical practice						
Surgery	1,547	894	57.8		1.00	
Internal medicine	2,643	1,542	58.3	p= 0.726	1.02	0.90 - 1.16
Number of working days						
Below 5	636	403	63.4		1.29	1.09 - 1.54
6 and over	3,554	2,033	57.2	p= 0.004	1.00	
Number of patients						
Less than 40	1,612	966	59.9		1.13	0.99 - 1.28
40 and over	2,578	1,470	57.0	p= 0.064	1.00	
Hours of consulting						
Less than 8	2,824	1,656	58.6		1.07	0.94 - 1.21
8 and over	1,366	780	57.1	p= 0.344	1.00	
4. Recreation						
Bedtime						
Before 12	3,040	1,887	62.1		1.79	1.56 - 2.06
After 12	1,150	549	47.7	p= 0.001	1.00	
Dulation of sleep						
-6 or 8+	1,028	623	60.6		1.00	
6 to 8	3,162	1,813	57.3	p= 0.065	0.87	0.76 - 1.01
Physical fatigue						
Aware	3,275	1,853	56.6		1.00	
Unaware	915	583	63.7	p= 0.001	1.35	1.16 - 1.57
Mental stress						
Aware	2,919	1,643	56.3		1.00	
Unaware	1,271	793	62.4	p= 0.001	1.29	1.13 - 1.48
5. Exercise						
Current habit of exercise						
No	2,094	1,144	54.6		1.00	
Yes	2,096	1,292	61.6	p= 0.001	1.33	1.18 - 1.51
6. Food intake						
Miso soup (Misoshiru)						
Less than daily	2,280	1,355	59.4		1.00	
Daily	1,910	1,081	56.6	p= 0.064	0.89	0.79 - 1.01
Japanese pickles (Tsukemono)						
Less than daily	2,005	1,224	61.0		1.00	
Daily	2,185	1,212	55.5	p= 0.001	0.80	0.70 - 0.90
Bread						
Less than daily	2,645	1,495	56.5		1.00	
Daily	1,545	941	60.9	p= 0.006	1.20	1.06 - 1.36
Milk						
Less than daily	2,533	1,428	56.4		1.00	
Daily	1,657	1,008	60.8	p= 0.004	1.20	1.06 - 1.36
Fresh vegetables						
Less than daily	1,543	790	51.2		1.00	
Daily	2,647	1,646	62.2	p= 0.001	1.57	1.38 - 1.78
Yellow and green vegetables						
Less than daily	2,530	1,371	54.2		1.00	
Daily	1,660	1,065	64.2	p= 0.001	1.51	1.33 - 1.72
Fruit						
Less than daily	2,083	1,099	52.8		1.00	
Daily	2,107	1,337	63.5	p= 0.001	1.56	1.37 - 1.76
7. Beverage						
Coffee						
Less than daily	3,036	1,902	62.6		1.00	
Daily	1,154	534	46.3	p= 0.001	0.51	0.45 - 0.59
Green tea						
Less than daily	645	426	66.0		1.00	
Daily	3,545	2,010	56.7	p= 0.001	0.67	0.57 - 0.80
Alcohol - frequency						
Less than daily	2,245	1,410	62.8		1.00	
Daily	1,945	1,026	52.8	p= 0.001	0.66	0.58 - 0.75
Alcohol - amount per day						
Under 1 go	2,433	1,540	63.3		1.00	
1 go and over	1,757	896	51.0	p= 0.001	0.60	0.53 - 0.68

p for chi-square was based on Mantel-Haenszel chi-square test.

Odds ratio and 95% CI were based on univariate unconditional logistic regression analysis.

The results of step-wise multivariate logistic regression analysis were shown in Table 4. By means of univariate logistic regression analysis, the following 17 factors were selected as statistically significant variables from the 22 variables for which significant odds ratios were recognized: female, age 50-59 years rather than under 50, BMI, peptic ulcer disease, health checkups, bedtime, mental fatigue or stress, regular physical exercise, consumption of fresh vegetables, consumption of yellow and green vegetables, consumption of fruit, heart disease, consumption of Japanese pickles, consumption of coffee, consumption of green tea, frequency of alcohol consumption, and amount of alcohol consumed.

**Table 4.** Multiple logistic regression analysis for smoking status (non-smoking).

Variables	Odds Ratio	95%CI
Body Mass Index		
Under 22	1.00	
22 and over	1.34	1.16 - 1.54
Heart disease		
No	0.67	0.48 - 0.94
Yes	1.00	
Peptic ulcer		
No	2.65	1.61 - 4.35
Yes	1.00	
Health check-ups		
No	1.00	
Yes	1.16	1.01 - 1.32
Bedtime		
Before 12	1.75	1.51 - 2.03
After 12	1.00	
Mental stress		
Aware	1.00	
Unaware	1.25	1.08 - 1.44
Current habit of exercise		
No	1.00	
Yes	1.35	1.18 - 1.54
Japanese pickles (Tsukemono)		
Not daily	1.00	
Daily	0.77	0.67 - 0.88
Fresh vegetables		
Not daily	1.00	
Daily	1.29	1.11 - 1.50
Yellow and green vegetables		
Not daily	1.00	
Daily	1.21	1.04 - 1.40
Fruit		
Not daily	1.00	
Daily	1.25	1.09 - 1.44
Coffee		
Not daily	1.00	
Daily	0.48	0.42 - 0.56
Green tea		
Not daily	1.00	
Daily	0.71	0.59 - 0.85
Alcohol - frequency		
Not daily	1.00	
Daily	0.82	0.70 - 0.97
Alcohol - amount per day		
Under 1 go	1.00	
1 go and over	0.71	0.60 - 0.84

Odds ratio and 95% CI were based on a step-wise multiple logistic regression analysis after adjusted for sex and age.

## DISCUSSION

### Physicians ought to be models of healthy living for those around them. Are they suitable subjects for such a study?

The valid response rate in the present study was much higher than those of previous surveys of physicians conducted in Japan^6^^,^^7^^,^^9^^-^^12^^)^. However, according to a survey of physicians conducted by the Ministry of Health and Welfare in 1982^30^^)^, the total physician population of Fukuoka Prefecture was 8,508, while the total number of the Fukuoka Prefecture Medical Association was 49%. Compared with the number of practicing physicians, for whom official figures of sex and age are available, the proportion of female physicians were smaller in study subjects ( 6% VS. 4%). Further, the proportions of subjects analyzed in the study were 60% for those in their 40s and over 80% for those of 50 and over, but only 11% of both male and female physicians under 40 years of age were subjects, those in their 20s being especially low. Since the percentage of physicians who smoke in these younger age groups is high^8^^)^, the actual prevalence of smoking among the physicians of Fukuoka Prefecture can be expected to be even higher than the present results. However, physicians in Japan, in particular the so-called “owner-practitioners”, who work independently in their own clinics, have roles both as models of healthy lifestyles for the local population and as educators who can directly persuade their patients to abandon the smoking habit^6^^)^. Among the subjects analyzed, those who are owner-practitioners number 3,154, and represent 97% of the total of 3,253 owner-practitioners in the prefecture. Although the number of employed physicians — many of whom are young — in the present study was too small to offer a clear understanding of their situation regarding smoking, the study succeeded in covering the vast majority of owner-practitioners. In this way, studies of this kind, where subjects are members of the Medical Association, are of significance.

### Effect of gender in the present study

One report^20^^)^ has suggested that an examination of the relationship between smoking and confounding factors by sex should be performed. In the present study, although the data was analyzed with males and females combined, no differences were observed when the analysis was carried out on the male data only.

### What was the current prevalence of smoking?

The proportions of smokers among the total number of the subjects whose data was analyzed in the present study were 43% for men and 9% for women. According to the National survey of circulatory disorders 1980, the prevalence of smoking among the general population in Japan were 63% in men and 10% in women. On the other hand, the age-adjusted percentages of smokers among physicians were 45% in males and 9% in females. The present prevalence of smoking among physicians in general is lower than that in the general population in Japan, and similar results have been found in the U.S. and the UK^10^^,^^13^^-^^15^^)^.

The highest prevalence of smoking in the subjects analyzed by age group in the present study was found in those under 40 years of age, followed by those in their 50s and 60s. A drop was seen in the rate of smokers actually in their 40s, while after the age of 50 the prevalence of smoking was found to decrease as the subjects grew older. This trend was seen even when male physicians were taken as a single category unto themselves. Generally, the prevalence of smoking tends to peak in the younger age groups their 20s and 30s, and thereafter to fall gradually with age^3^^)^. However, the results of the present study showed that the prevalence of smoking among physicians in their 40s was lower than in their 50s and 60s. The proportion of female physicians showed no significant difference dependent on age. On the other hand, the proportion of employed physicians in their 40s was significantly lower than those in their 50s and 60s: this difference in age groups is thought to be the primary factor in the low level of smoking in physicians in their 40s. Moreover, the proportion of non-smoking physicians in their 40s who had never smoked was significantly higher than that of such physicians in their 50s and 60s, and was similar to the proportion found in the under-40 age group. The age when physicians who smoked started smoking was reported to be in their late teens and early 20s^9^^)^. Study results of the hazardous effects of smoking on health were published after the mid-1950s^31^^)^. It appears that the high rate of non-smokers in their 40s in the present study was brought about by the fact that they were in their late teens and early 20s, when they had been studying in the medical school, during the mid-1950s.

### The relationship between the individual characteristics of physicians and their smoking status

Owner-physicians, both male and female, had a higher prevalence of smoking than employed physicians. This tendency has been reported in recent studies, but the reasons for it are unclear^12^^)^.

Specialty of medical practice was broadly divided into surgical and internal medicine, but no correlation was found. In a study in Chiba Prefecture^9^^)^ and a spot-check survey among the members of die Japan Medical Association^11^^)^, it was pointed out that the prevalence of smoking in internists was lower than that in surgeons. It is thought that the reason for this discrepancy was that, because nearly 80% of the subjects who participated in the present study operated their own clinics, the styles of medical practice between the two groups did not differ so much that their lifestyles were affected.

A significant correlation was seen between a shorter work week and a non-smoking lifestyle. A survey among industrial workers revealed that there was no correlation between either hours of overtime work or amount of free time and actual quitting of smoking^19^^)^. In the case of physicians, a work week consisting of fewer days of medical practice is sometimes seen among older physicians who entrust work to their successors, and this was presumed to have a marked effect. In practice, this factor was not selected as a statistically significant variable in a step-wise multivariate regression analysis after adjustment for sex and age.

### Consideration of lifestyle in relation to a physician’s smoking status using the study variables

In the present study, non-smokers had a higher BMI than smokers, and showed a tendency toward obesity. A positive dose-response relationship was reported in smokers between the amount of smoking and body weight, but light smokers (smoking less than 20 cigarettes a day) had a lower BMI than non-smokers^21^^)^. In the present study, many of the responses to the question on the amount of smoking were incomplete, and so analysis by amount of smoking was not done. However, the present results were seemed to reflected that there were few heavy smokers among the subjects of the survey.

In the results of a survey of physicians in Chiba Prefecture, more than 40% of subjects gave, as the reason for giving up smoking, some illness^9^^)^. In the present study, those with heart disease tended not to smoke, and those with peptic ulcer disease tended to smoke. Further prospective studies are required to ascertain whether the smoking behavior is a cause or a result of the illness.

This study included variables that indicated correlations with smoking in previous reports, and examined their relationship with a current non-smoking status. In those earlier studies, the following were seen as characteristics of smokers: no regular exercise, little consumption of vegetables and fruit, and consumption of salty foods, coffee, and alcohol^16^^,^^18^^,^^20^^,^^22^^-^^26^^)^ and the present study yielded similar findings. However, it was reported that the consumption of green tea and milk showed different relationships with the smoking habit, depending on sex and age. In the present study the results of the consumption of green tea and milk were seen to tend to resemble those of middle-aged and older men^18^^,^^20^^)^.

Fruit in Japan is customarily eaten for the dessert course of a meal or between meals. This manner of fruit consumption may take part in forming a trade-off relationship with smoking status. Moreover, in view of the pattern of activities during a physician’s day, coffee or green tea is frequently used to punctuate the daily routine. To accompany this with smoking in such circumstances is sometimes called “indulgent smoking”, and many physicians tend to follow this behavior. Such “indulgent smokers” do not inhale large quantities of smoke, and, as in the case of BMI, this relationship could be due to the fact that heavy smoking is relatively rare among physicians^32^^)^.

The above findings suggest that the smoking physician smokes relatively small amounts of smoke. Among light smokers, greater success in achieving prolonged abstinence and a lower relapse rate after smoking cessation are reported^33^^)^. For these reasons, smokers who are physicians could be assumed, as a group, to be more successful in programs designed to help participants to give up the habit. On the other hand, one study has reported that physicians, who already have considerable medical knowledge about smoking, cannot be expected to have a high success rate in programs offering just that knowledge^34^^)^. In view of the characteristics of physicians, it would appear to be important for a smoking physician, and for any smoking cessation program aimed at such a subject, to establish a healthy, health-conscious lifestyle including such elements as increased consumption of vegetables and fruit and an appropriate pattern of alcohol intake. Prospective studies will now be required to further clarify the relationship between these variables and the habit of smoking.
